
JOURNAL INFORMATION
==============================
NLM Title Abbreviation: Cancer Med
Journal ID: Cancer Med
NLM Journal ID: 101595310
Journal ID: CAM4

Cancer Medicine

EISSN: 2045-7634
Publisher: Wiley

ARTICLE INFORMATION
==============================
PMCID: PMC11474529
DOI: 10.1002/cam4.4877
Article ID: CAM44877
Article version: 1
Subjects: Issue Information, Issue Information

Issue Information

Electronic publication date: 2023 Aug 31
Publication date: 2023 Aug
Volume: 12
Issue: 15
First page: 15763
Last page: 15768
Copyright: © 2023 The Authors. Cancer Medicine published by John Wiley & Sons Ltd.
License: This is an open access article under the terms of the http://creativecommons.org/licenses/by/4.0/ License, which permits use, distribution and reproduction in any medium, provided the original work is properly cited.
License URL: https://creativecommons.org/licenses/by/4.0/

Figure count: 0
Table count: 0
Page count: 6

source-schema-version-number: 2.0
cover-date: August 2023
details-of-publishers-convertor: Converter:WILEY_ML3GV2_TO_JATSPMC version:6.4.9 mode:remove_FC converted:15.10.2024

==============================

Cancer Medicine

Open Access

### Aims and Scope

*Cancer Medicine* is a peer-reviewed, open access, interdisciplinary journal providing rapid publication of cutting-edge research from global biomedical researchers across the cancer sciences. The journal considers submissions from all oncologic specialties, including, but not limited to, the following areas:

### Cancer biology

Molecular biology • cellular biology • molecular genetics • genomics • immunology • epigenetics • metabolic studies • proteomics • cytopathology • carcinogenesis • drug discovery and delivery

### Clinical cancer research

Translational research • clinical trials • chemotherapy • radiation therapy • surgical therapy • clinical observations • clinical guidelines • genetic consultation • ethical considerations

### Cancer prevention

Behavioral science • psychosocial studies • screening • nutrition • epidemiology and prevention • community outreach

### Bioinformatics

Gene expressions profiles • gene regulation networks • genome bioinformatics • pathway analysis • prognostic biomarkers

*Cancer Medicine* publishes original research articles, systematic reviews, meta-analyses, and research methods papers, along with invited editorials and commentaries. Original research papers must report well-conducted research with conclusions supported by the data presented in the paper.

We aim to be a truly global forum for high-quality cancer research, and we think that the best research should be published and made widely accessible as quickly as possible. *Cancer Medicine* publishes papers submitted directly to the journal and those referred from a select group of prestigious journals published by Wiley-Blackwell.

*Cancer Medicine* is a Wiley Open Access journal, one of a new series of peer-reviewed titles publishing quality research with speed and efficiency. For further information visit the Wiley Open Access website at http://www.wileyopenaccess.com.

### Open Access and Copyright

All articles published by *Cancer Medicine* are fully open access: immediately freely available to read, download and share. All articles accepted from 14 August 2012 are published under the terms of the Creative Commons Attribution License. All articles accepted before this date were published under a Creative Commons Attribution Non-Commercial License. The Creative Commons Attribution License permits use, distribution and reproduction in any medium, provided the original work is properly cited and allows the commercial use of published articles. Copyright on any research article in a journal published by *Cancer Medicine* is retained by the author(s). Authors grant Wiley a license to publish the article and identify itself as the original publisher. Authors also grant any third party the right to use the article freely as long as its integrity is maintained and its original authors, citation details and publisher are identified. Further information about open access license and copyright can be found at http://www.wileyopenaccess.com/details/content/12f25db4c87/Copyright--License.html.

### Purchasing Print Reprints

Print reprints of Wiley Open Access articles can be purchased from corporatesales@wiley.com.

### Disclaimer

The Publisher and Editors cannot be held responsible for errors or any consequences arising from the use of information contained in this journal; the views and opinions expressed do not necessarily reflect those of the Publisher or Editors, neither does the publication of advertisements constitute any endorsements by the Publisher or Editors of the products advertised.

Wiley Open Access articles posted to repositories or websites are without warranty from Wiley of any kind, either express or implied, including, but not limited to, warranties of merchantability, fitness for a particular purpose, or non-infringement. To the fullest extent permitted by law Wiley disclaims all liability for any loss or damage arising out of, or in connection with, the use of or inability to use the content.

### Editor-in-Chief

Stephen Tait, PhD
*Institute of Cancer Sciences, University of Glasgow*
*Glasgow, UK*

### Co-Editors

Alfred Lam, PhD
*Griffith University*
*Queensland, Australia*

Maryam Lustberg, MD
*Yale University School of Medicine*
*New Haven, CT, USA*

### Deputy Editors

Qian Chen, PhD
*John Wiley & Sons*
*Beijing, China*

Sumit Sharma, PhD
*John Wiley & Sons*
*Copenhagen, Denmark*

Radhika N. Shridharan, PhD
*John Wiley & Sons*
*Toronto, Canada*

### Founding Editor

Qingyi Wei, MD, PhD
*Duke Cancer Institute*
*Duke University Medical Center*
*Durham, NC, USA*

### Associate Editors

### Clinical Cancer Research

Ichiro Abe, PhD
*Fukuoka University Chikushi Hospital*
*Fukuoka, Japan*

Stefano Arcangeli, MD
*University of Milan Bicocca*
*Rome, Italy*

Wendy Chan Wing Lok, MBBS
*Department of Clinical Oncology*
*The University of Hong Kong*
*Hong Kong SAR, China*

Dhyan Chandra, PhD
*Roswell Park Cancer Institute*
*Buffalo NY, USA*

Ahmet Emre Eşkazan, MD
*Istanbul University-Cerrahpaşa*
*Istanbul, Turkey*

Lei Huang, PhD
*National Institutes of Health*
*Bethesda, MD, USA*

Zhaohui Huang, PhD
*Wuxi Cancer Institute*
*Affiliated Hospital of Jiangnan University*
*Jiangsu Province, China*

Hirotaka Ishida, PhD, MD
*Tohoku University*
*Sendai, Japan*

Farhadul Islam, PhD
*Rajshahi University*
*Rajshahi, Bangladesh*

Julia Ismael, MD
*Director, Gedyt Foundation*
*Buenos Aires City, Argentina*

Victor C. Kok, MD, PhD, FACP
*Director, Cancer Center of Kuang Tien General Hospital*
*Shalu Taichung, Taiwan*

Weidong Le, MD, PhD
*Sichuan Academy of Medical Sciences*
*Sichuan Provincial Hospital*
*Chengdu, Sichuan, China*

Shenglong Li, PhD
*Department of Bone and Soft Tissue Surgery*
*Cancer Hospital of China Medical University*
*Liaoning Cancer Hospital & Institute*
*Shenyang, China*

Allison Quick, MD
*The Ohio State University Comprehensive Cancer Center*
*Columbus, OH, USA*

Hemamali Samaratunga, FRCPA
*Aquesta Specialised Uropathology*
*University of Queensland*
*Queensland, Australia*

Nicole C. Schmitt, MD
*Winship Cancer Institute*
*Emory University*
*Atlanta, GA, USA*

Rashmi Kanagal-Shamanna, MD
*The University of Texas MD Anderson Cancer Center*
*Houston, TX, USA*

Takeshi Terashima, MD, PhD
*Advanced Preventive Medical Sciences Research Center*
*Kanazawa University*
*Department of Gastroenterology*
*Kanazawa University Hospital*
*Kanazawa, Japan*

Diane Von Ah, PhD, RN
*The Ohio State University*
*Columbus, OH, USA*

Jing Wang, MD, PhD
*Department of Breast Surgical Oncology*
*National Clinical Research Center for Cancer*
*Cancer Hospital, Chinese Academy of Medical Sciences and Peking Union Medical College*
*Beijing, China*

Song Xu, MD, PhD
*Department of Lung Cancer Surgery*
*Tianjin Medical University General Hospital*
*Tianjin, China*

Shinichi Yamashita, PhD
*Fukuoka University Chikushi Hospital*
*Fukuoka, Japan*

Ka Yu Tse, MBBS, PhD
*The University of Hong Kong*
*Hong Kong SAR, China*

Dan P. Zandberg, MD
*UPMC Hillman Cancer Center*
*Pittsburgh, PA, USA*

Yan Zhao, PhD
*First Affiliated Hospital of Xi'an Jiaotong University*
*Xi'an, China*

Zhenjian Zhuo, PhD
*Peking University Shenzhen Graduate School*
*Shenzhen, China*

### Cancer Biology

Juan-Carlos Acosta, PhD
*University of Edinburgh*
*Edinburgh, UK*

Florian Bock, PhD
*Maastricht University*
*Maastricht, Netherlands*

Lisa Bouchier-Hayes, PhD
*Baylor College of Medicine*
*Houston, TX, USA*

Dipanjan Chanda, PhD
*Department of Endocrinology*
*Kyungpook National University Hospital*
*Daegu, South Korea*

Jean Ehrland-Ricci, PhD
*INSERM*
*Nice, France*

Nashwa El-Khazragy
*Ain Shams University*
*Cairo, Egypt*

Trivadi Sundaram Ganesan, MD, PhD
*Sri Ramachandra Institute of Higher Education and Research*
*Chennai, India*

Vignir Helgason, PhD
*University of Glasgow*
*Institute of Cancer Sciences*
*Glasgow, UK*

Gabriel Ichim, PhD
*INSERM*
*Nice, France*

Gemma Kelly, PhD
*WEHI*
*Melbourne, Australia*

Jennifer Morton, PhD
*Beatson Institute*
*Glasgow, UK*

Nathiya Muthalagu, PhD
*Indian Institute of Technology*
*Chennai, India*

Victor Peperzak, PhD
*Universitair Medisch Centrum Utrecht*
*Utrecht, Netherlands*

Cristina Muñoz-Pinedo, PhD
*IDIBELL*
*Barcelona, Spain*

Triona Ni Chonghaile, PhD
*Royal College of Surgeons Ireland*
*Dublin, Ireland*

Kristopher Sarosiek, PhD
*Harvard University*
*Cambridge, MA, USA*

Ajay Singh, PhD
*Mitchell Cancer Institute*
*University of South Alabama*
*Mobile, AL, USA*

Wenge Zhu, PhD
*The George Washington University School of Medicine and Health Science*
*Washington, DC, USA*

### Cancer Prevention

Peter Baade, PhD
*Cancer Council Queensland*
*Queensland, Australia*

Guangfu Jin, PhD
*Nanjing Medical University*
*Nanjing, China*

Gilberto Morgan, MD
*Founder - OncoAlert*
*Skåne University Hospital*
*Lund, Sweden*

Matthew B. Schabath, PhD
*H. Lee Moffitt Cancer Center and Research Institute*
*Tampa, FL, USA*

Kyle M. Walsh, PhD
*Duke University Medical Center*
*Durham, NC, USA*

### Bioinformatics and Biostatistics

Stephan Bernhart, PhD
*Leipzig University*
*Leipzig, Germany*

Juan Cui, PhD
*University of Nebraska-Lincoln*
*Lincoln, NE, USA*

Martin Fischer, PhD
*Leibniz Institute on Aging – Fritz Lipmann Institute (FLI)*
*Jena, Germany*

Quinn Ostrom, PhD, MPH
*Duke University School of Medicine*
*Durham, NC, USA*

Ping Zhang, PhD
*Griffith University*
*Queensland, Australia*

### Reviewing Editors

Dr. Zubing Mei
*Anorectal Disease Institute / Shuguang Hospital*
*Shanghai, China*

Dr. Ahmed Sadik
*German Cancer Research Center (DKFZ)*
*Heidelberg, Germany*

Dr. Chandrani Sarkar
*University of South Alabama*
*Mobile, AL, USA*

Dr. Kai Zhang
*University of Kentucky College of Medicine*
*Lexington, KY, USA*

### Editorial Board Members

### Clinical Cancer Research

Yung-Jue Bang
*Seoul National University College of Medicine*
*Seoul, Korea*

Anthony Chan
*The Chinese University of Hong Kong*
*Hong Kong, China*

Ke-Neng Chen
*Peking University School of Oncology*
*Beijing, China*

Jiuwei Cui
*The First Hospital of Jilin University*
*Jilin, China*

Giuseppe Di Lorenzo
*Ospedale Pagani*
*ASL Salerno, Italy*

Peter J. Fuller
*Prince Henry Institute*
*Victoria, Australia*

Maura L. Gillison
*Ohio State University Comprehensive Cancer Center*
*Columbus, OH, USA*

Hao Guo
*Dana Farber Cancer Institute*
*Boston, MA, USA*

Dong Hua
*Affiliated Hospital of Jiangnan University*
*Wuxi, China*

Yeul Hong Kim
*Korea University College of Medicine*
*Seoul, Korea*

Guido Kroemer
*University of Paris Descartes*
*Paris, France*

Lalit Kumar
*All India Institute of Medical Sciences*
*New Delhi, India*

Razelle Kurzrock
*UCSD Moores Cancer Center*
*San Diego, CA, USA*

Hongyu Li
*Department of Gastroenterology of Shenyang Northern Hospital*
*Shenyang, China*

Geoffrey Liu
*Princess Margaret Hospital*
*Toronto, Canada*

Xiaowen Liu
*Fudan University Shanghai Cancer Center*
*Fudan, China*

Jiade J. Lu
*Shanghai Proton and Heavy Ion Center*
*Shanghai, China*

Li Mao
*University of Maryland*
*Baltimore, MD, USA*

Akira Nakagawara
*Chiba University*
*Chiba, Japan*

Jingxuan Pan
*Sun Yat-sen University Zhongshan Ophthalmic Center*
*Guangzhou, China*

Pamela L. Paris
*University of California*
*San Francisco, CA, USA*

Chao-Nan (Miles) Qian,
*Sun Yat-sen University Cancer Center*
*Guangzhou, China*

Dimitrios H. Roukos
*Ioannina University School of Medicine*
*Ioannina, Greece*

Ajay Singh
*University of South Alabama*
*Mitchell Cancer Institute*
*Mobile, AL, USA*

Pankaj K. Singh
*University of Nebraska Medical Center*
*Omaha, NE, USA*

Frank A. Sinicrope
*Mayo Clinic*
*Rochester, MN, USA*

Benjamin D. Smith
*The University of Texas MD Anderson Cancer Center*
*Houston, TX, USA*

Jun Wang
*BGI-Shenzhen*
*Shenzhen, China*

Senming Wang
*Southern Medical University*
*Guangzhou, China*

Glen J. Weiss
*Translational Genomics Research Institute*
*Phoenix, AZ, USA*

Xiaohua Wu
*Fudan University Shanghai Cancer Center*
*Shanghai, China*

Li Yan
*GlaxoSmithKline Pharmaceuticals*
*Pennsylvania, USA*

Xianglin Yuan
*Huazhong University of Science and Technology*
*Wuhan, China*

Rongyu Zang
*Fudan University Zhongshan Hospital*
*Shanghai, China*

Sheng Luo
*Duke University Medical Center*
*Durham, NC, USA*

Dinggang Li
*LUDAOPEI Hematology Hospital*
*Beijing, China*

Ninghan Feng
*The Affiliated Wuxi No.2 People's Hospital of Nanjing Medical University*
*Wuxi, China*

### Cancer Biology

Richard J. Ablin
*University of Arizona College of Medicine*
*Tucson, AZ, USA*

Dolores Cahill
*University College Dublin*
*Dublin, Ireland*

David W. Chan
*The University of Hong Kong*
*Hong Kong, China*

Ming Chen
*Department of Pathology*
*Duke University School of Medicine*
*Durham, NC, USA*

Zhijun Dai
*Second Affiliated Hospital of Xi'an Jiaotong University*
*Xi'an, China*

Rosalind Eeles
*Institute of Cancer Research*
*Sutton, UK*

Manel Esteller
*Bellvitge Biomedical Research Institute (IDIBELL)*
*Barcelona, Spain*

**Kai Fu**
*Central South University*
*Hunan, China*

**Elizabeth A. Grimm**
*The University of Texas MD Anderson Cancer Center*
*Houston, TX, USA*

**Xiaoxiang Guan**
*Nanjing University School of Medicine*
*Nanjing, China*

**Jos Jonkers**
*Netherlands Cancer Institute*
*Amsterdam, The Netherlands*

**Zhiyong Han**
*The George Washington University*
*Washington, DC, USA*

**Giseli Klassen**
*Federal University of Parana*
*Curitiba, Brazil*

**Chuan-Yuan Li**
*Duke University Medical Center*
*Durham, NC, USA*

**Gang Li**
*University of British Columbia*
*Vancouver, Canada*

**Kiven E. Lukong**
*University of Saskatchewan*
*Saskatoon, Canada*

**Youyong Lu**
*Beijing Institute for Cancer Research*
*Beijing, China*

**Yuichi Machida**
*Mayo Clinic*
*Rochester, MN, USA*

**Kenneth Offit**
*Memorial Sloan-Kettering Cancer Center*
*New York, NY, USA*

**Athanasios G. Papavassiliou**
*University of Athens Medical School*
*Athens, Greece*

**Kenneth Pienta**
*University of Michigan*
*Ann Arbor, MI, USA*

**Jack A. Schalken**
*Radboud University Medical Centre*
*Nijmegen, Netherlands*

**Takeshi Senga**
*Nagoya University Graduate School of Medicine*
*Nagoya, Japan*

**Shi-Yong Sun**
*Emory University School of Medicine and Winship Cancer Institute*
*Atlanta, GA, USA*

**Kazu Ushijima**
*National Cancer Center*
*Tokyo, Japan*

**David Vaux**
*Walter and Eliza Hall Institute of Medical Research*
*Parkville, Australia*

**Fengyi Wan**
*John Hopkins University*
*Baltimore , MD, USA*

**Meilin Wang**
*Nanjing Medical University*
*Nanjing, China*

**Ping Pui Wong**
*Barts Cancer Institute*
*Queen Mary University of London*
*London, UK*

**Keping Xie**
*The University of Texas MD Anderson Cancer Center*
*Houston, TX, USA*

**Gong Yang, D.V.M.,Ph.D**
*Shanghai Medical School*
*Fudan University*
*Shanghai, China*

**Ke-Da Yu**
*Fudan University Shanghai Cancer Center*
*Shanghai, China*

**Li Zhang**
*The University of Texas MD Anderson Cancer Center*
*Houston, TX, USA*

**Xiang Zhou**
*Institutes of Biomedical Sciences and Shanghai Cancer Center*
*Fudan University*
*Shanghai, China*

### Cancer Prevention

**Nadine Barrett**
*Duke University School of Medicine*
*Durham, NC, USA*

**Marianne Berwick**
*University of New Mexico*
*Albuquerque, NM, USA*

**Eduardo Cazap**
*Latin-American & Caribbean Society of Medical Oncology*
*Buenos Aires, Argentina*

**Kexin Chen**
*Tianjin Medical University Cancer Institute and Hospital*
*Tianjin, China*

**Steve Dubinett**
*David Geffen School of Medicine at UCLA*
*Los Angeles, CA, USA*

**Kari Hemminki**
*German Cancer Research Center*
*Heidelberg, Germany*

**Zhibin Hu**
*Nanjing Medical University*
*Nanjing, China*

**Stephen D. Hursting**
*University of North Carolina*
*Chapel Hill, NC, USA*

**Wei-Hua Jia**
*Sun Yat-Sen University Cancer Center*
*Guangzhou, China*

**Daehee Kang**
*Seoul National University College of Medicine*
*Seoul, Republic of Korea*

**Lambertus A.**
*Kiemeney, Radboud University Medical Centre*
*Nijmegen, Netherlands*

**Qing Lan**
*National Cancer Institute*
*Bethesda, MD, USA*

**Donghui Li**
*The University of Texas MD Anderson Cancer Center*
*Houston, TX, USA*

**Guojun Li**
*The University of Texas MD Anderson Cancer Center*
*Houston, TX, USA*

**Jiachun Lu**
*Guangzhou Medical University*
*Guangdong, China*

**Hung N. Luu**
*University of South Florida*
*Tampa, FL, USA*
*Vanderbilt University School of Medicine*
*Nashville, TN, USA*

**Hongxia Ma**
*Nanjing Medical University School of Public Health*
*Nanjing, China*

**Somdat Mahabir**
*National Cancer Institute*
*Bethesda, MD, USA*

**Girish Maru**
*ACTREC Tata Memorial Centre*
*Navi Mumbai, India*

**Xiaoping Miao**
*Huazhong University of Science and Technology*
*Wuhan, China*

**Andrew Olshan**
*University of North Carolina*
*Chapel Hill, NC, USA*

**Jong Y. Park**
*H. Lee Moffitt Cancer Center & Research Institute*
*Tampa, FL, USA*

Susan K. Peterson
*The University of Texas MD Anderson Cancer Center*
*Houston, TX, USA*

Thomas E. Rohan
*Albert Einstein College of Medicine*
*Bronx, NY, USA*

Michael E. Scheurer
*Baylor College of Medicine*
*Houston, TX, USA*

Sheng Wei
*Huazhong University of Science and Technology*
*Wuhan, China*

Momiao Xiong
*The University of Texas Health Science Center*
*Houston, TX, USA*

Jianfeng Xu
*Northshore University Health System*
*Evanston, IL, USA*

Xue Qin Yu
*Cancer Council NSW & The University of Sydney's School of Medicine*
*Sydney, Australia*

Hongping Yu
*Guangxi Medical University Cancer Hospital/Guangxi Cancer Center*
*Guangxi, China*

ZiYuan Zhou
*Third Military Medical University, College of Public Health*
*ChongQing, China*

### Editorial Advisory Board

Laurence Baker
*University of Michigan*
*Ann Arbor, MI, USA*

Webster K. Cavenee
*Ludwig Institute for Cancer Research*
*New York, NY, USA*

David Christiani
*Harvard School of Public Health*
*Boston, MA, USA*

Robert B. Diasio
*Mayo Clinic*
*Rochester, MN, USA*

B. Mark Evers
*Lucille P. Markey Cancer Center*
*Lexington, KY, USA*

Ernest Hawk
*The University of Texas MD Anderson Cancer Center*
*Houston, TX, USA*

Leroy Hood
*Institute for Systems Biology*
*Seattle, WA, USA*

Scott M. Lippman
*University of California*
*San Diego, CA, USA*

Dongxin Lin
*Chinese Academy of Medical Sciences*
*Beijing, China*

James Mulé
*H. Lee Moffitt Cancer Center & Research Institute*
*Tampa, FL, USA*

Philip Agop Philip
*Wayne State University*
*Detroit, MI, USA*

Steven R. Patierno
*Duke University Medical Center*
*Durham, NC, USA*

Hongbing Shen
*Nanjing Medical University*
*Nanjing, China*

Nicholas Vogelzang
*Comprehensive Cancer Centers of Nevada*
*Las Vegas, NV, USA*

Cun-Yu Wang
*UCLA School of Dentistry*
*Los Angeles, CA, USA*

Hongyang Wang
*Eastern Hepatobiliary Surgery Institute / Hospital*
*Shanghai, China*

The cover image is based on the Research Article *Clinical Analysis of EV-Fingerprint to Predict Grade Group 3 and Above Prostate Cancer and Avoid Prostate Biopsy* by Adrian Fairey et al., https://doi.org/10.1002/cam4.6216.
